# Dissociation between arithmetic relatedness and distance effects is modulated by task properties: An ERP study comparing explicit vs. implicit arithmetic processing

**DOI:** 10.1016/j.biopsycho.2014.10.003

**Published:** 2014-12

**Authors:** Chiara Avancini, Giovanni Galfano, Dénes Szűcs

**Affiliations:** a Department of Psychology, University of Cambridge, Cambridge, UK; b Dipartimento di Psicologia dello Sviluppo e della Socializzazione, Università di Padova, Padova, Italy; c Centro di Neuroscienze Cognitive, Università di Padova, Padova, Italy

**Keywords:** ERPs, Mental arithmetic, Arithmetic relatedness, Distance effect, Magnitude representation

## Abstract

Event-related potential (ERP) studies have detected several characteristic consecutive amplitude modulations in both implicit and explicit mental arithmetic tasks. Implicit tasks typically focused on the arithmetic relatedness effect (in which performance is affected by semantic associations between numbers) while explicit tasks focused on the distance effect (in which performance is affected by the numerical difference of to-be-compared numbers). Both task types elicit morphologically similar ERP waves which were explained in functionally similar terms. However, to date, the relationship between these tasks has not been investigated explicitly and systematically. In order to fill this gap, here we examined whether ERP effects and their underlying cognitive processes in implicit and explicit mental arithmetic tasks differ from each other. The same group of participants performed both an implicit number-matching task (in which arithmetic knowledge is task-irrelevant) and an explicit arithmetic-verification task (in which arithmetic knowledge is task-relevant). 129-channel ERP data differed substantially between tasks. In the number-matching task, the arithmetic relatedness effect appeared as a negativity over left-frontal electrodes whereas the distance effect was more prominent over right centro-parietal electrodes. In the verification task, all probe types elicited similar N2b waves over right fronto-central electrodes and typical centro-parietal N400 effects over central electrodes. The distance effect appeared as an early-rising, long-lasting left parietal negativity. We suggest that ERP effects in the implicit task reflect access to semantic memory networks and to magnitude discrimination, respectively. In contrast, effects of expectation violation are more prominent in explicit tasks and may mask more delicate cognitive processes.

## Highlights

• ERPs were recorded while performing number matching and arithmetic verification tasks.
• Access to the arithmetic facts lexicon is modulated by task properties.
• Arithmetic relatedness and distance effects are sensitive to task properties.
• Matching tasks involve semantic processes.
• Verification tasks involve semantic and detection of mismatch processes.

## 1 Introduction

Recent years have witnessed significant progress in understanding how mental arithmetic is performed by the human brain (Jasinski & Coch, 2012; for a review see Arsalidou & Taylor, 2011). Several crucial insights were provided by event-related brain potential (ERP) studies. Most studies capitalized on two behavioral paradigms: the arithmetic-verification task (e.g., Ashcraft and Battaglia, 1978, Winkelman and Schmidt, 1974), in which arithmetic knowledge is explicitly required by the task, and the number-matching task (LeFevre, Bisanz, & Mrkonjic, 1988), in which arithmetic knowledge is accessed implicitly. In both paradigms, participants are presented with a sequence of stimuli. First, two numbers are presented (hereafter, the ‘cues’) typically with an arithmetic symbol in between. Second, another number (hereafter, the probe) is presented. Participants performing an arithmetic-verification task decide whether the probe is the correct result of the arithmetic operation stated by the cue pair or not. Participants performing a number-matching task simply judge whether the probe is one of the numbers displayed in the cue pair or not. These paradigms are thought to elicit morphologically and functionally similar ERP phenomena. However, the cognitive operations required by them can be thought to be substantially different, so to date it is not clear whether ERP phenomena and related cognitive processes are indeed similar in these tasks because previous ERP studies invariably focused on a single paradigm. In order to resolve this controversy, here we report a study in which the same participants took part in both implicit and explicit tasks. We analyzed high spatial density ERP data in order to decide unequivocally whether ERP phenomena can be interpreted in functionally similar terms in these tasks.

We used the ERP correlates of two well-known phenomena related to semantic and lexical components in number processing, i.e. the arithmetic relatedness effect and the numerical distance effect. The arithmetic relatedness effect has been demonstrated in the arithmetic-verification task. Participants are slower in rejecting the probe when this is the correct result to a table-related problem as compared to a false but unrelated result (e.g., Stazyk et al., 1982, Winkelman and Schmidt, 1974). This effect has been attributed to the fact that arithmetic facts are stored in memory in a network-like structure whose mechanisms are similar to those postulated for semantic networks in the linguistic domain (Ashcraft, 1992). Such network organization allows the automatic retrieval of incorrect but related answers especially in the case of multiplication and addition, although the latter to a much lesser extent (Roussel, Barrouillet, & Fayol, 2002). The arithmetic relatedness effect has also been shown in number-matching tasks in that participants are slower in rejecting non-matching probes when these are either the correct result (e.g., De Brauwer and Fias, 2009, Rusconi et al., 2004, Rusconi et al., 2006, Thibodeau et al., 1996) or a table-related result with respect to the numbers in the cue pair (e.g., Galfano et al., 2003, Zamarian et al., 2006). The distance effect was originally discovered in magnitude comparison tasks (e.g., Moyer and Landauer, 1967, Restle, 1970) and it refers to the fact that performance is better (more accurate and faster) when the proposed false probe and the (not shown) correct answer differ by a large as opposed to small distance. The distance effect has only been investigated in arithmetic-verification tasks in both behavioral (Ashcraft and Battaglia, 1978, Restle, 1970) and ERP experiments (Szűcs and Csépe, 2004a, Szűcs and Csépe, 2004b, Szűcs and Csépe, 2005). In these experiments, the probes of the critical conditions were false answers deviating by a small (hereafter small distance) or large amount (hereafter large distance) from the correct answer. The difference in behavioral and electrophysiological responses elicited by small and large distance probes has been interpreted as the distance effect.

With regard to the arithmetic-verification paradigm, Niedeggen and Rösler (1999) (also see Niedeggen, Rösler, & Jost, 1999) examined multiplication fact retrieval and manipulated both the degree of relatedness of false results to correct results and whether false results were numerically close or far to the correct solution (numerical distance). They argued that a probe representing an incorrect solution would be processed similarly to semantic violations which elicit the so-called N400 effect (Kutas & Hillyard, 1980; for review see Kutas & Federmeier, 2011). Indeed, ERPs time-locked to probe onset revealed an N400-like effect with a more pronounced negativity for false probes in comparison to correct probes (also see Szűcs & Soltész, 2010). In addition, consistent with the arithmetic relatedness effect, participants were slower in rejecting probes consisting of a false but table-related result than probes arithmetically unrelated to the cue numbers. Probes corresponding to unrelated solutions were associated with significantly more negative N400 amplitudes than probes representing related solutions at centro-parietal electrode sites. Distance of probes with respect to the correct solution did not affect N400 amplitude. In sharp contrast, the subsequent ERP component, the P300 or P3b, which is typically postponed in arithmetic as compared to other types of tasks (e.g., Galfano et al., 2011, Niedeggen et al., 1999), was sensitive to both arithmetic relatedness and distance. The amplitude of the P3b increased as a probe deviated numerically from the correct solution. In addition, it was always larger for probes consisting of unrelated than related solutions.

Niedeggen and Rösler (1999) (also see Niedeggen et al., 1999) argued that N400 amplitude was inversely related to the amount of spreading activation from the cue numbers, whereas P3b amplitude more generally reflected (im)plausibility of probes, consistent with the view that the P3b is inversely related to the subjective probability of stimuli (Donchin & Coles, 1988).

More recently, Szűcs and Csépe (2005) have focused on the distance effect and reported that larger deviations from correct solutions resulted in an enhanced negativity of an early wave peaking at around 270 ms (also see Szűcs and Csépe, 2004a, Szűcs and Csépe, 2004b). This component seemed to resemble the so called N2b (for a review see, e.g., Folstein & Van Petten, 2008), which is considered to be a general correlate of mismatch detection independent of numerical information (e.g., Hsu and Szűcs, 2011, Szűcs et al., 2007, Wang et al., 2000). Importantly, when analyzing ERPs in the time window of the N400 effect reported by Niedeggen and Rösler (1999), it was found that the amplitude of the difference potentials (incorrect minus correct probes) was not modulated as a function of distance. Szűcs and Csépe (2005) concluded that the N2b and the N400 have different functional roles and later demonstrated that they might in fact be dissociated based on their different scalp topography (Szűcs et al., 2007). The N2b appears in averaged potentials over frontal scalp sites whereas the N400 effect is typically observed in the difference potentials over central and more posterior scalp sites. Regarding the P3b, distance seems to affect both its amplitude and latency. Probes corresponding to larger deviations from the correct solutions elicit a larger and delayed P3b in comparison to probes more distant from the correct solutions (also see Jasinski and Coch, 2012, Núñez-Peña and Escera, 2007).

With regard to the number-matching paradigm, Galfano et al. (2003) tested how table related probes and neutral probes affected behavioral data in a number-matching task. They found that related probes elicited longer reaction times than neutral probes as a function of automatic spreading of activation in the arithmetic facts lexicon. Interestingly, the same pattern emerged when the magnitude and the distance between the product of the cue digits of the neutral probes was smaller than the magnitude and the distance of the related probes. This suggested that the impact, if any, of the distance effect may be rather weak in implicit number-matching tasks, at least at the behavioral level. Galfano, Mazza, Angrilli, and Umiltà (2004) demonstrated an N400 effect similar to that reported by Niedeggen and Rösler (1999), the effect was time-locked to probe onset even when arithmetic knowledge was irrelevant to the task at hand. Non-matching, arithmetically neutral, probes were responded to faster and elicited a relative larger negativity than non-matching arithmetically related probes. The N400 effect peaked at around 380 ms after probe onset and was most pronounced over frontal and central scalp regions. Galfano, Penolazzi, Vervaeck, Angrilli, and Umiltà (2009) used a paradigm in which non-matching probes could either be the product of both cue numbers (strong cue-probe association), the multiple of one of the cue numbers (weak cue-probe association), or an arithmetically neutral number with respect to the cue numbers (no cue-probe association). Performance in the non-matching conditions was significantly better when the probe number was arithmetically neutral compared to when it was arithmetically related, either strongly (i.e., the product) or weakly (i.e., a multiple of either cue numbers), to the cue digits. ERP data confirmed the N400 effect reported by Galfano et al. (2004). At around 400 ms after probe onset, the amplitude of the N400 was larger for arithmetically neutral and multiple probes than for product probes, while the potentials elicited by multiple probes were close to those elicited by neutral probes. Interestingly, an analysis focusing on an earlier time window centered at around 275 ms time-locked to the probe onset revealed that product and multiple probes elicited a similar and larger relative negativity than arithmetically neutral probes. Galfano et al. (2009) interpreted this pattern as evidence that automatic activation in the network of multiplication facts spreading from cue numbers fades away more quickly for weakly associated items (i.e., multiple probes) than for strongly associated items (i.e., product probes). These ERP effects seemed to be widespread over the scalp.

The ERP effects discussed above most probably reflect the consecutive activation of several, potentially overlapping, cognitive processes. ERP has millisecond time resolution which is able to discriminate between quickly succeeding effects, allowing to directly observe the neural activity which immediately follows stimulus presentation (Luck, Woodman, & Vogel, 2000). Furthermore, different ERP components have been linked to qualitatively different cognitive processes as, for example, magnitude processing (Núñez-Peña, Cortiñas, & Escera, 2006), semantic processing (Kutas & Federmeier, 2011), mismatch detection (Bennett, Duke, & Fuggetta, 2014), target detection (Mulert et al., 2004).

In the present study, we aimed to shed light on critical issues raised by the studies discussed above. First, as noted, we aimed to clarify whether ERP data in explicit and implicit arithmetic tasks using multiplications can be interpreted in functionally similar terms. Unlike previous studies, here, for the first time, both implicit number-matching and explicit arithmetic-verification tasks were administered to the same participants using the same stimuli.

The second aim of our study (a consequence of the first aim) was to investigate the relationship, if any, between the arithmetic relatedness and the distance effects and their sensitivity to task requirements using a high-density ERP system. The arithmetic relatedness effect seems to be associated with an N400-like component in both arithmetic-verification (e.g., Niedeggen & Rösler, 1999) and number-matching tasks (e.g., Galfano et al., 2009). In contrast, to the best of our knowledge, the distance effect has never been investigated in the context of implicit arithmetic tasks, whereas in the arithmetic-verification task, such a phenomenon is well reflected at the level of the N2b (Szűcs & Csépe, 2005) and the P3b (Niedeggen and Rösler, 1999, Szűcs and Csépe, 2005). It is worth noting that the ERP modulation reported by Galfano et al. (2009) for the arithmetic relatedness effect was temporally very similar to the N2b modulation reported by Szűcs and Csépe (2005) for the distance effect. Here, we also aimed to shed light on whether the two modulations are somehow related to each other. One possibility is that the distance and the arithmetic relatedness effects are reflected in two negative components that are independent of each other (or even in components with same polarity and time course but different topography, see Hsu & Szűcs, 2011). The data reported by Szűcs and Csépe (2005) and Galfano et al. (2009) seem to be consistent with the latter scenario (numerical distance resulting in an earlier amplitude modulation of ERPs, and arithmetic relatedness resulting in a later modulation). Unfortunately, a topographic comparison of the two effects was precluded by the fact that Galfano et al. (2009) used an electrode setting with low spatial resolution. In the present study, we overcame this issue, by using a high-density 129-channel system.

Third, the consecutive appearance of an N2b and an N400 ERP wave are dominant features of ERPs in verification tasks and several studies based their experimental hypotheses on these waves. Hence, we aimed to clarify whether the N2b and the N400 are elicited irrespective of whether arithmetic and numerical knowledge are accessed explicitly and are relevant for the task at hand. On the one hand, under the widely shared assumption that the N400 reflects automatic spreading activation (e.g., Galfano et al., 2004, Niedeggen and Rösler, 1999, Szűcs and Csépe, 2005, Szűcs and Soltész, 2010), we predicted that this component would be evident in both arithmetic-verification and number-matching tasks. In contrast, if the N2b is sensitive to expectation violation rather than to (semantic) magnitude processing, this component should primarily appear in the verification task where arithmetic knowledge is accessed explicitly.

In this study we focused on multiplication. This was because arithmetic facts are thought to be stored in memory in a network-like structure, whose mechanisms would be similar to those of the semantic networks in the linguistic domain (Ashcraft, 1992). Such network organization allows the automatic retrieval of incorrect but related answers especially in the case of multiplication and addition, although the latest to a lesser extent (e.g., Roussel, Barrouillet, & Fayol, 2002). On the other hand, quantitative knowledge and calculus strategies are instead mainly used for solving subtractions and divisions (Dehaene & Cohen, 1997; Zhou et al., 2006). Hence, overall, multiplication seems the best task to use for comparing numerical semantic memory processes and the associated N400 ERP signal in both explicit and implicit tasks. In addition, multiplication tasks also seem to elicit a larger N400 as compared to addition (e.g., Jasinski and Coch, 2012, Zhou et al., 2006) and so far ERP studies on number-matching tasks have only examined this arithmetic operation (e.g., Galfano et al., 2004). Hence, using multiplication also enabled us to connect to the previous findings more clearly.

## 2 Method

### 2.1 Participants

Twenty-two participants took part in the experiment in Cambridge, UK. All participants gave their informed consent prior to their inclusion in the study. Six participants were excluded from further analyses because their EEG signal was contaminated by artifacts in more than 25% of their total trials. Our final sample thus consisted of 16 participants (7 females) aged from 20 to 31 (*M* = 25.06). They had normal or corrected-to-normal vision and had no history of brain injury or mental illness. The study was approved by the Psychology Research Ethics Committee at the University of Cambridge, UK.

### 2.2 Number-matching task

#### 2.2.1 Stimuli

The stimuli appeared in white against a black background and were displayed centered on a 17-inch Apple LCD monitor placed about 50 cm in front of the participants. Each character appeared in font 40. The distance between the two numbers in the cue was 80 pixels. Each trial consisted of a number pair cue (e.g., “3” and “9”) followed by a number probe (e.g., “27”). The task of the participants was to decide whether the probe number matched either number in the cue. Half of the stimuli were matching trials, half were non-matching trials. There were different experimental conditions based on cue-probe association types (see Table A.1). For the non-matching conditions (i.e., the relevant trials to test our hypotheses), there were product trials, multiple trials, small distance neutral trials, large distance neutral trials, and non-matching filler trials, occurring with the same frequency. The same two single-digit numbers served as the cue for product, multiple, small distance, and large distance trials (e.g., “3·9”). For product trials, the probe was the product of the two digits in the cue (e.g., “27”). For multiple trials, the probe was the multiple of either cue digits (e.g., “63”). Unlike standard number-matching paradigms (e.g., Galfano et al., 2003), we used two types of arithmetically neutral trials. In the small distance condition, the probes deviated ±2 units from the product of the cue digits. In the large distance condition, the probes deviated ±4 units from the product of the cue digits. These latter two categories of trials allowed us to estimate the distance effect.

The final category of non-matching trials consisted of filler trials. The cue and the probe of the filler trials included a double-digit number (e.g., “26·7” and “45”), in order to have trials in which the participants saw double-digit numbers in the cue. These trials also served to balance the number of related and unrelated trials in the non-matching condition. However, because they played no role in assessing our hypotheses, they were not included in the analyses (also see, e.g., Galfano et al., 2009, Thibodeau et al., 1996).

Unlike the non-matching stimuli, on matching trials, one of the digits in the cue matched the probe (see Table A.1). These trials, not relevant for addressing arithmetic relatedness and distance effects, also included five categories. The first four categories served to balance the different types of probes in the non-matching trials: *probe-balancing related to the product* trials, *probe-balancing related to the multiple* trials, *probe-balancing related to the small distance* trials, *probe-balancing related to large distance* trials. The final category of matching trials consisted of cue-balancing trials and had the same cue as the non-matching crucial conditions. It is important to note that these trials were not analyzed as they were not relevant to assess our hypotheses and were also likely to be contaminated by stimulus repetition effects.

In the crucial conditions (i.e., non-matching product, multiple, small distance, and large distance trials), several criteria for stimulus selection were adopted in order to minimize the occurrence of confounds. Cues consisting in ties (e.g., “7·7”) were not included in the stimulus set, because they seem to have a privileged memory access compared to other problems (e.g., Ashcraft and Battaglia, 1978, Campbell and Gunter, 2002). Combinations of cues and probes that might have elicited activation on the basis of arithmetic relations other than multiplication (e.g., subtraction, “8·3”, and “5”) were also discarded from the stimulus set. Cues containing “0” or “1” were also discarded from the stimulus set, because problems including these numbers as operands are more likely to be solved by activating rules rather than arithmetic facts (e.g., Jost et al., 2004, McCloskey et al., 1991). In order to have a reasonable amount of stimuli, we had to include probes with “5” or “0”, which were equally present in all crucial conditions. Importantly, the average magnitude of the probes was similar in each critical condition. Finally, each probe in the critical conditions appeared the same number of times.

With all these constraints taken into account, we selected a list of 5 stimuli for each category of both matching and non-matching items. One stimulus out of five had a partial match between one of the numbers in the cue and the probe. However, this was true for every condition. The stimuli were presented in 9 blocks of 84 trials each. In each block, there were 50 non-matching trials and 34 matching trials. This imbalance had the purpose of maximizing the number of trials useful for assessing our hypotheses (matching trials are irrelevant to assess arithmetic relatedness and distance effects) and allowed us to obtain a potential of 90 data points for each critical cell of the design. The specific ratio between matching and non-matching trials was chosen in order to prevent that participants subjectively perceive incorrect trials more rarely than correct trials. According to Donchin (1981), the amplitude of the P3b is sensitive not only to absolute proportions of task-relevant stimulus categories but also to perceived subjective difference between proportions of categories, moreover Szűcs and Csépe (2005) demonstrated that the amplitude of the P3b is modulated by the proportion of correct answers, while no effect was found on the amplitude of the negative components between 230 and 330 ms and between 240 and 400 ms. In light of those results, Szűcs and Soltész (2010), in an arithmetic verification task, decreased the proportion of correct trials to 40% in order to decrease the perceived subjective probability. The study replicated the same N400 effect found in a previous experiments where the proportion of correct answers was 50%. On the other hand the amplitude of the P3b decreased suggesting an influence of subjective probability. Therefore, having a higher proportion of incorrect trials allowed us to rule out the effect of subjective probability on the amplitude of the P3b without influencing the amplitude of the N2b and of the N400.

The sequence of trials was randomized separately for each participant.

#### 2.2.2 Procedure

**Fig. 1:**
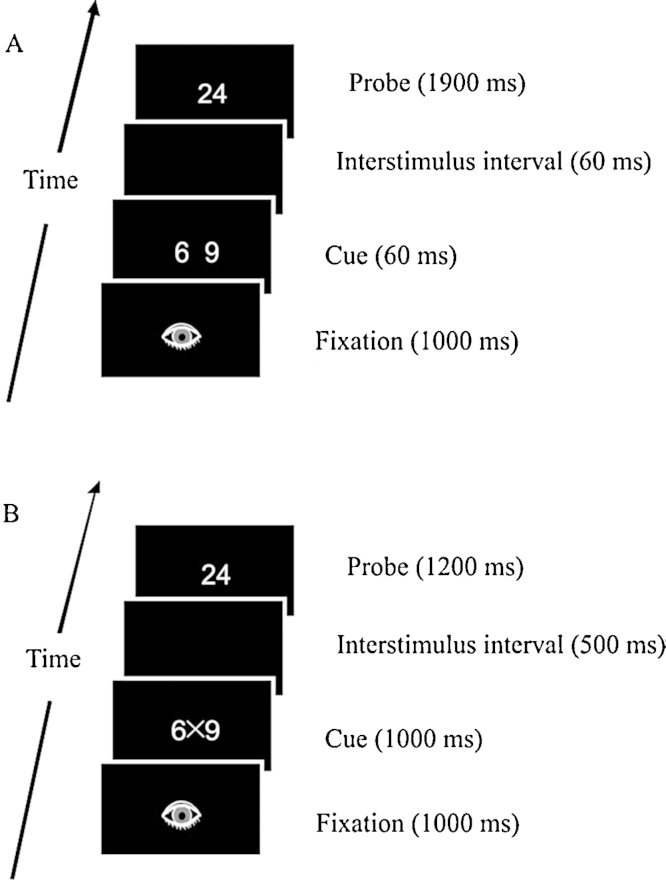
Panel A. Sequence of events in the number-matching task. A non-matching multiple trial is illustrated. Stimuli are not drawn to scale. Panel B. Sequence of events in the arithmetic verification task. A trial with a false but related (i.e., multiple) result is illustrated. Stimuli are not drawn to scale.

Testing took place in a sound-attenuated, electrically shielded, dimly-lit room. Before the experiment started, participants performed some practice trials until they became familiar with the task. The sequence of events is illustrated in Fig. 1A. Each trial began with a fixation point (the picture of an eye) shown for 1000 ms at the center of the screen. The cue digits were presented synchronous to fixation point offset. The cue frame lasted 60 ms and was followed by an interstimulus interval (ISI) of 60 ms, resulting in a fixed 120-ms stimulus onset asynchrony (SOA), which is best suited for capturing arithmetic relatedness effects with both products and multiples in number-matching tasks (e.g., Galfano et al., 2003, Thibodeau et al., 1996). Then, the probe appeared and remained visible for 1900 ms, irrespective of the participants’ response. The participants responded by means of a gamepad, using their index fingers. They were required to press the right button when the probe matched either number of the cue, and the left button when there was no match. Response key assignment was counterbalanced across participants. The instructions emphasized both speed and accuracy. Participants were allowed to take short breaks between blocks, and were instructed to execute eye-blinks during the fixation frame only. Both the number-matching task and the arithmetic-verification task were performed in the same testing session with a short break in between. The number-matching task was always performed first because, in order to assess the difference between explicit and implicit tasks, it was critical to ensure that arithmetic remained task-irrelevant during the number-matching task and to minimize the transfer of task set between the two tasks. In other words, keeping such order allowed us to avoid the occurrence of carryover effects which might have favored the possibility that participants *deliberately* activated arithmetic knowledge also when performing number matching, i.e., a task in which arithmetic knowledge should be accessed, if any, only implicitly as it is not relevant to task requirements.

Behavioral data were analyzed using median response times (RTs) for correctly responded trials and the percentage of correct responses. A one-way ANOVA was carried out with probe type as factor. In order to test the arithmetic relatedness effect, we conducted pairwise comparisons between the product condition and the arithmetically neutral condition (i.e., an average score collapsing small distance and large distance probes), and between the product condition and the multiple condition. The effect of numerical distance was measured by means of pairwise comparisons between the small distance condition and large distance condition.

### 2.3 Arithmetic-verification task

#### 2.3.1 Stimuli

The stimuli appeared on a 17-inch Apple LCD monitor in white on a black background. Each trial consisted of a cue pair with the multiplication sign in between, followed by the equal sign (e.g. “3 × 9 =”). The cue pairs were the same used in the number-matching task (see Table A.2). The cue was followed by a probe (e.g. “27”). The task was to decide whether the probe was the correct result of the multiplication previously displayed or not. There were 4 different experimental conditions based on the cue-probe association types. The probe could be either the correct result (i.e., product probe trial), or a false result. The latter included related results (i.e., multiple probe trials), unrelated small distance probe trials, and unrelated large distance probe trials. Stimuli for all these types of trials were the same as those used in the number-matching task. There were 4 blocks of 100 trials each. In each block there were 25 trials for each condition. This imbalance allowed us to obtain a potential of 100 data points for each critical cell of the design. The proportion between correct and incorrect answer was chosen in order to prevent that participants subjectively perceive incorrect results more rarely than correct results (e.g., Szűcs and Csépe, 2005, Szűcs and Soltész, 2010). The sequence of trials was randomized separately for each participant.

#### 2.3.2 Procedure

This was the same as in the number-matching task with the following exceptions. The cue frame lasted 1000 ms and was followed by a 500-ms ISI. The probe remained visible for 1200 ms (see Fig. 1 Panel B). These timing variations were necessary because here the task explicitly required participants to use arithmetic (i.e., unlike the number-matching task, here arithmetic was task-relevant). Participants were required to press the right button when the probe was the correct result of the multiplication stated in the cue, and the left button when it was not. Response key assignment was counterbalanced across participants. Behavioral data were analyzed using median RTs for correctly responded trials and percentage of correct responses. An ANOVA was carried out with the probe types as factors. The product probes, unlike in the matching task, were not included in the ANOVA because they were responded to with a different response key with respect to other probes. In order to test the effect of arithmetic relatedness, we conducted pairwise comparisons between the multiple condition and the arithmetically neutral condition (i.e., an average score collapsing the small distance and the large distance conditions). The effect of numerical distance was measured by means of pairwise comparisons between the small distance condition and the large distance condition.

### 2.4 EEG recording and analysis

**Fig. 2:**
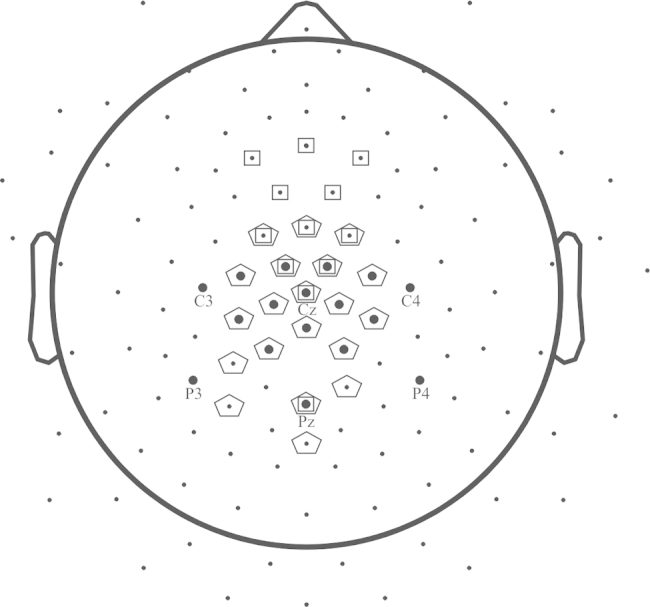
The dots set represents the 129 Geodesic channel net. Bold dots show the electrodes used to detect the latency of the N400 effect; empty squares show the electrodes used to detect the latencies of the N2b; empty pentagons show the electrodes used to detect the latencies of the P3b. The N400 effect was detected at 350–450 ms time window at electrodes 7, 30, 31, 37, 54, 55, 79, 80, 87, 105, 106, C3, P3, C4, P4, Cz e Pz; N2b was detected at 200–350 ms time window at electrodes 4, 5, 6, 7, 13, 19, 106, 112, Cz, Pz and Fz; P3b peak latency was detected at 250–600 ms time window over electrodes 6, 7, 13, 30, 31, 37, 53, 54, 55, 60, 72, 78, 79, 80, 87, 105, 106, 112, Cz and Pz.

Electric brain activity was recorded by means of a 129-channel Geodesic sensor net (Electrical Geodesics, Oregon) (see Fig. 2). Impedance was kept below 10 kΩ. The sampling rate was 500 Hz. An anti-aliasing low-pass filter of 70 Hz was applied during data acquisition. Offline, the data were band-pass filtered between 0.01 and 30 Hz, baseline-corrected relative to the −100 to 0 ms interval before cue onset, and recomputed to an average reference. For the number-matching task, the continuous EEG was segmented into epochs between 1000 ms and 3020 ms after fixation point onset. For the arithmetic-verification task, the continuous EEG was segmented into epochs between 1000 ms and 3700 ms after fixation point onset. Spline interpolation was carried out on individual channels if required. Epochs were excluded from analysis if they met any of the following artifact rejection criteria: voltage deviations exceeded ±100 μV relative to baseline, the maximum gradient exceeded 50 μV, or activity was lower than 0.5 μV. Across participants, 78.94% of trials and 75.16% of the trials were retained after filtering, artifact rejection and topographical interpolation for number matching and arithmetic verification, respectively. For both tasks, the accepted epochs were averaged for each of the crucial conditions and then difference averages were computed by subtracting the product condition to the other three condition.

We used two complementary approaches. First, in the verification task we had clear expectations about the expected component structure of ERPs. Hence, ERP components of interest were identified in averaged data in time windows based on reported latencies in the literature (e.g., Domahs et al., 2007, Galfano et al., 2004, Jasinski and Coch, 2012, Jost et al., 2004, Núñez-Peña and Suárez-Pellicioni, 2012, Zhang et al., 2003). Peak latencies and peak amplitudes were measured as the most positive or most negative data point in a time window. Components were detected on electrodes where their maximum could be expected according to the literature. Electrode locations and electrode numbers are shown in Fig. 2. The P3b (most positive peak) and the N2b (most negative peak) were detected in averaged ERP between 250 and 600 ms after probe onset and between 200 and 350 ms after probe onset, respectively. The peaks of the N400 were detected in incorrect minus correct difference waves between 350 and 450 ms after probe onset.

From the above data, the effects of arithmetic relatedness and numerical distance on the peak latencies and peak amplitudes were tested by repeated measures analyses of variance (ANOVAs). One-way ANOVAs were conducted with probe type as a four-level factor (*product* vs. *multiple* vs. *small distance* vs. *large distance*); the ANOVAs conducted on the ERP difference waveforms had probe type as a three-level factor (*multiple* minus *product*, *small distance* minus *product*, *large distance* minus *product*). The effect of arithmetic relatedness was measured by means of pairwise comparisons between the product condition and the arithmetically neutral conditions (the small distance and the large distance conditions), between the product condition and multiple condition, and between the multiple and the neutral conditions (the small distance and the large distance conditions). The effect of numerical distance was measured by means of pairwise comparisons between the small distance condition and large distance condition. The N400 effect was detected at 350–450 ms time window at electrodes 7, 30, 31, 37, 54, 55, 79, 80, 87, 105, 106, C3, P3, C4, P4, Cz and Pz (see Fig. 2).

**Fig. 3:**
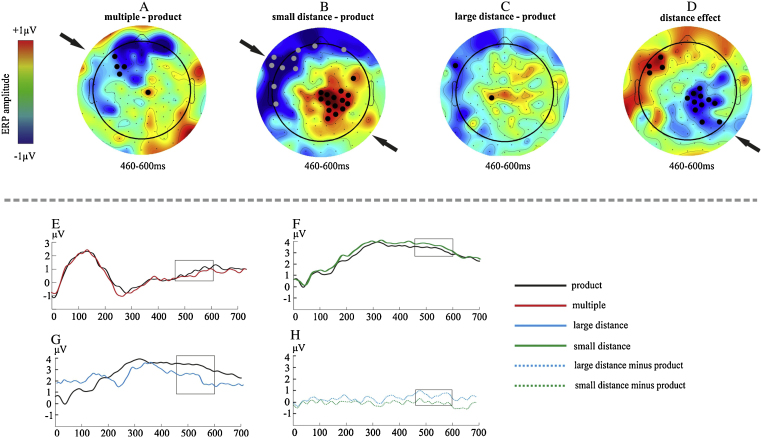
Interpolated scalp maps in the matching task. The topography of multiple minus product, small distance minus product and large distance minus product are shown (A–C). The fourth topography (D) shows the distance effect. Electrodes with significant effects are marked by bold dots (*p* < 0.005). Arrows point to significant differences between conditions. The bottom part of the figure shows the grand averages from significant electrodes that are marked by bold dots in the first part of the figure. The box highlights the time window where the effects were found in the above topography. (E) Mean ERPs elicited by product and multiple probes (electrodes pooled: 26. 27, 28, F3); (F) Mean ERPs elicited by small distance and product probes (electrodes pooled: Cz, 55, 77, 78, 79, 80, 85, 86, 87, P4, 93, 98, 103, C4); (G) Mean ERPs elicited by large distance and product probes (electrode pooled: 38); (H) Difference potentials showing the distance effect (electrodes pooled: 55, 78, 79, 80, 84, 86, 87, 93, T6, 98, 105). Note that positive voltage is upwards.

**Fig. 4:**
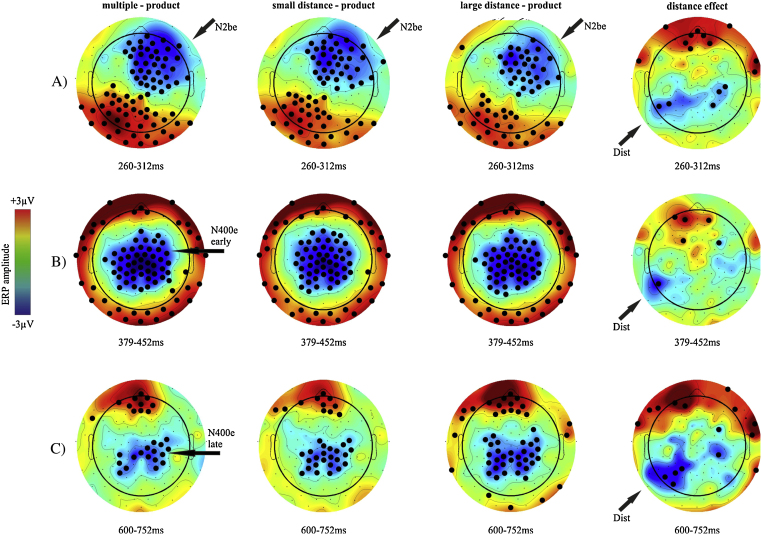
Interpolated scalp maps in the verification task. The topography of incorrect minus correct proposed results are shown in the first three columns; the fourth column shows the distance effect given by the difference between small distance and large distance. Electrodes with significant effects are marked by bold dots (*p* < 0.005). Arrows point to the N2b effect, the earlyN400 effect (N400e early); the late N400 effect (N400e late) and to the distance effect (Dist). The N400 effects are only marked in column 1 but they are also present in columns 2 and 3.

A second analysis approach examined ERP amplitude by point-by-point within-participant ANOVAs. Time intervals where statistical effects reached significance (*p* < 0.005) over a minimum of 15 consecutive sampling points at least at 6 electrode channels simultaneously (see headmaps in Fig. 3, Fig. 4) were considered to demonstrate significant effects (see, e.g., Szűcs & Soltész, 2010). This conservative criterion was adopted to control for Type I error. Pairwise comparisons (Bonferroni corrected) were carried out between each condition in the intervals found to show significant condition effects by the above ANOVAs. Brain Vision Analyzer, Matlab 7.1 and SPSS were used for performing all analyses.

## 3 Results

### 3.1 Number-matching task

#### 3.1.1 Behavioral data

Behavioral performance was assessed by median RTs for correctly responded trials and percentage of correct responses (see Table 1). Matching stimuli and non-matching filler stimuli were not analyzed, because they served no purpose for addressing either arithmetic relatedness or distance effects.

**Table 1:** Mean values and standard errors (in brackets) for median reaction times (in ms) and percentage of correct responses as a function of probe type in the number-matching task and in the arithmetic-verification task.

	Number-matching task		Arithmetic-verification task	
	RT	% correct	RT	% correct
Product	678 (35)	92 (2.2)	595 (24)	93 (1.4)
Multiple	657 (33)	95 (2.2)	587 (23)	95 (0.9)
Small distance	658 (35)	93 (2.1)	606 (25)	95 (1.2)
Large distance	670 (42)	96 (1.7)	583 (22)	97 (0.7)
Neutral	664 (38)	94 (1.8)	594 (23)	96 (0.8)

A one-way ANOVA conducted on median RTs with probe type as a four-level factor (product vs. multiple vs. small distance vs. large distance) revealed that the main effect was close to significance (*F*(3,45) = 2.451, *p* = 0.07, *η*_*p*_^2^ = 0.14). The effect of the arithmetic relatedness was measured by means of pairwise comparisons between the product condition and the arithmetically neutral conditions, and between the product condition and the multiple condition. Participants rejected a neutral probe (*M* = 664 ms, SE = 38) significantly faster than product probes (*M* = 678 ms, SE = 35). Interestingly, multiple probes (*M* = 657 ms, SE = 33) were responded to significantly faster than product probes, and they were not statistically different when compared to neutral probes (*p* = 0.35). Hence, unlike Galfano et al. (2009), performance for multiple probes was more similar to performance for neutral probes rather than to performance for product probes. One possibility is that this may reflect the impact of factors related to stimuli selection, given that the current study used different selection criteria. The effect of numerical distance was measured by means of pairwise comparisons between the small distance condition and the large distance condition. No statistical difference was found (*p* = 0.27).

An ANOVA on the percentage of correct responses also showed a trend toward a significant main effect of probe type (*F*(3,45) = 2.62, *p* = 0.06, *η*_*p*_^2^ = 0.15). Pairwise comparisons showed that participants responded significantly more accurately to neutral probe (*M* = 94%, SE = 1.8) than to product probes (*M* = 92%, SE = 2.2), consistent with RT data. No other comparisons relevant for testing arithmetic relatedness and distance effects were statistically significant. This pattern confirms that no speed-accuracy tradeoff affected the present data.

#### 3.1.2 ERP data

ERPs were assessed by means of one-way ANOVAs and pairwise comparisons conducted on both latencies and amplitude (see Table 2 for mean latencies and Fig. 3 for the results on the amplitudes).

**Table 2:** Mean values and standard errors (in brackets) for P3b, N2b and N400 latencies in both number matching task and arithmetic verification task.

	Product	Multiple	Small dist.	Large dist.	Neutral
Matching task P3b	439 ms (14)	439 ms (15)	425 ms (15)	451 ms (16)	438 ms (15)
Verification task P3b	445 ms (11)	500 ms (13)	474 ms (14)	484 ms (14)	479 ms (14)
Matching task N2b	255 ms (5)	253 ms (5)	261 ms (6)	263 ms (5)	262 ms (5)
Verification task N2b	285 ms (7)	296 ms (4)	297 ms (5)	286 ms (7)	291 ms (5)

		Multiple – product	Small distance – product	Large distance – product	Neutral
Matching task N400		398 ms (3)	396 ms (3)	397 ms (3)	397 ms (2)
Verification task N400		404 ms (4)	401 ms (4)	401 ms (3)	401 ms (3)

As regards latencies, in the N400 time window, no effect of probe type was found (*p* = 0.9). In the N2b window, a significant main effect of probe type was found (*F*(3,45) = 3.24, *p* = 0.031, *η*_*p*_^2^ = 0.18). Paired *t*-tests showed a statistically significant difference between the product and neutral conditions (*t*(15) = −2.78, *p* = 0.014) and between the multiple and the neutral conditions (*t*(15) = −2.71, *p* = 0.016). Latencies were longer in the neutral condition (*M* = 261.00 ms, SE = 5) than in the product condition (*M* = 255.27 ms, SE = 5) and were longer in the neutral condition than in the multiple condition (*M* = 253.35 ms, SE = 5).

In the P3b time window the main effect of probe type was significant (*F*(3,45) = 3.42, *p* = 0.025), *η*_*p*_^2^ = 0.19). Only the distance effect was significant (*t*(15) = −2.93, *p* = 0.010) with large distance probes (*M* = 450.71 ms, SE = 15) eliciting longer latencies than small distance probes (*M* = 424.63 ms, SE = 15).

Amplitudes were analyzed by means of point-by-point ANOVAs (see Szűcs & Soltész, 2010). There was an overall effect of probe type between 460 and 600 ms (*F*(3,45) ≥ 4.89, *p* ≤ 0.005). Contrasts (Tukey-corrected *p* for all conditions: *p* ≤ 0.005) showed a long-lasting effect with amplitudes in the multiple, small distance and large distance conditions being less positive over left-frontal electrodes than in the product condition. Amplitudes at right central-posterior electrodes were instead more positive for the multiple, small distance and large distance conditions than for the product condition. Moreover, contrasts between the large distance and the small distance conditions revealed a distance effect. Indeed, large distance probes elicited less positive amplitudes at right central-posterior electrodes and more positive amplitudes at frontal left electrodes than small distance probes.

### 3.2 Arithmetic-verification task

#### 3.2.1 Behavioral data

Behavioral performance was assessed by median RTs for correctly responded trials and percentage of correct responses (see Table 1). Product probes were not included in the ANOVA because, unlike the number-matching task, in the arithmetic-verification task they were responded to with a different response key with respect to other probes. A one-way ANOVA conducted on median RTs with probe type as factor (multiple vs. small distance vs. large distance) revealed that the main effect was statistically significant (*F*(2,30) = 8.949, *p* = 0.001, *η*_*p*_^2^ = 0.39). The effect of arithmetic relatedness was measured by means of pairwise comparisons between the multiple condition and the arithmetically neutral conditions. Participants took a similar time for rejecting multiple probes (*M* = 587 ms, SE = 23) and arithmetically neutral probes (*M* = 594 ms, SE = 23). Hence, RTs showed apparently no evidence for the arithmetic relatedness effect. Importantly, however, a clear distance effect emerged, as participants were significantly faster in rejecting large distance probes (*M* = 583 ms, SE = 22) than small distance probes (*M* = 606 ms, SE = 25).

An ANOVA on the percentage of correct responses also showed a significant main effect of probe type, (*F*(3,30) = 3.43, *p* = 0.04, *η*_*p*_^2^ = 0.19). Participants responded significantly more accurately to large distance probes (*M* = 97%, SE = 0.7) than to small distance probes (*M* = 95%, SE = 1.2), consistent with RT data. No other comparisons relevant for testing our hypotheses were statistically significant. This pattern confirms that no speed-accuracy tradeoff affected the present data.

#### 3.2.2 ERP data

**Fig. 5:**
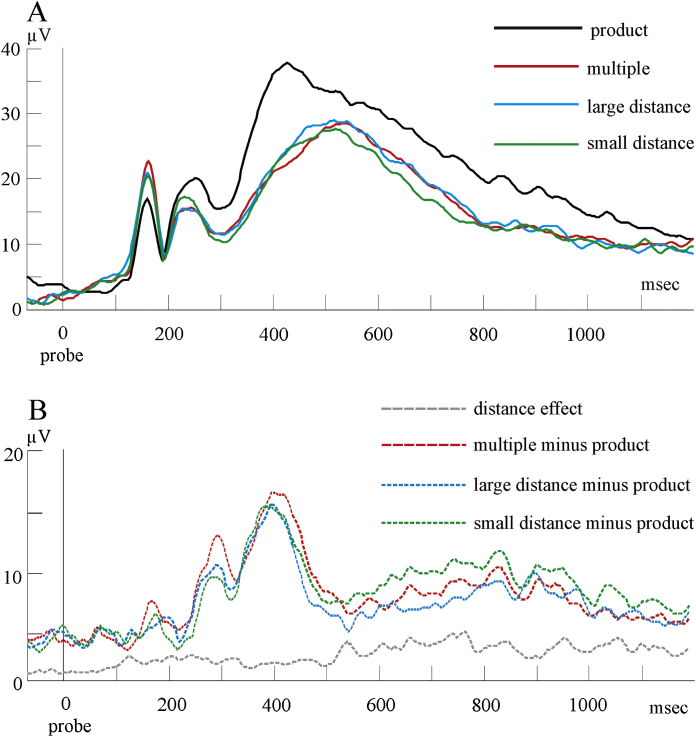
Global field power in the verification task (A), incorrect minus correct ERPs in the verification task (B). Note that positive is upwards.

ERPs were assessed by means of one-way ANOVAs and pairwise comparisons conducted on both latencies and amplitude. Mean amplitude topographies are shown in Fig. 4, global field power is shown in Fig. 5 and mean latencies are shown in Table 2.

As regards latencies, in the N400 time window, no effect of probe type was found (*p* = 0.31). In the N2b window, the main effect of probe type was significant (*F*(3,45) = 3.72, *p* = 0.038, *η*_*p*_^2^ = 0.20). Paired t-tests showed a significant difference between the product and the multiple conditions (*t*(15) = −2.11, *p* = 0.053) with longer latency in the multiple condition (*M* = 296.44, SE = 4) than in the product condition (*M* = 284.56, SE = 7). The distance effect emerged (*t*(15) = 2.09, *p* = 0.054) with longer latency in the small distance condition (*M* = 296.46, SE = 5) than in the large distance condition (*M* = 286.27, SE = 7).

P3b latency showed a significant probe type effect (*F*(3,45) = 8.03, *p* = 0.002, *η*_*p*_^2^ = 0.35). Paired *t*-tests showed statistical differences between the multiple and the neutral conditions (*t*(15) = 2.72, *p* = 0.016), between the product and the multiple conditions (*t*(15) = −3.79, *p* = 0.002) and between the product and the neutral conditions (*t*(15) = −2.371, *p* = 0.032). Latency was longer in the multiple condition (*M* = 499.60, SE = 13) than in the neutral condition (*M* = 478.86, SE = 14), was longer in the multiple condition than in the product condition (*M* = 445.43, SE = 11) and it was longer in the multiple condition than in the neutral condition.

**Fig. 6:**
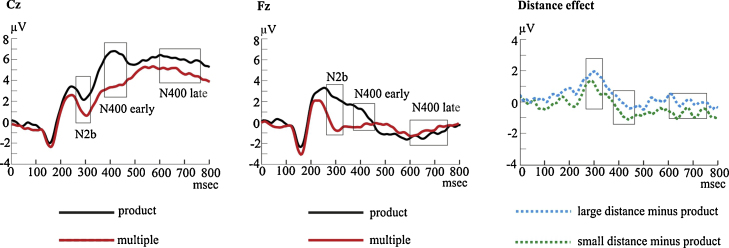
The verification task. The two leftmost panels show mean ERPs in the product and multiple conditions elicited at Cz and Fz locations during the verification task. The rightmost figure represents the distance effect in difference potentials. The boxes highlight the time windows shown in the topography in Fig. 4. Note that positive voltage is upwards.

Results of the point-by-point analysis of the amplitude data are shown in Fig. 4 and the time course of events is illustrated in Fig. 6. Significant effects of probe type emerged in 5 consecutive time windows: 160–200 ms, 260–312 ms, 379–452 ms, 600–752 ms and 752–952 ms (*F*(3,45) ≥ 4.89; *p* ≤ 0.005). In the 260–312 ms time window, the multiple, the small distance, and the large distance conditions elicited less positive amplitudes over right central-anterior electrodes and more positive amplitudes over left posterior electrodes than product probe types (Tukey-corrected p for all conditions: *p* ≤ 0.005). We identified the effect between 379 and 452 ms as a typical centro-parietal N400-like effect. Amplitudes were less positive over central electrodes in multiple, small distance and large distance than in product condition. We identified the effect between 600 and 752 ms as a late N400 effect because its scalp topography and amplitude range was very similar to the preceding N400-like effect. In this time window the multiple, the small distance and the large distance conditions showed less positive amplitude than the product condition over central posterior electrodes. Finally, there was a distance effect in the 260–312 ms, 379–452 ms, 600–752 ms and 752–952 ms time windows: amplitudes were less positive over left posterior electrodes and more positive over frontal electrodes in the large distance condition than in the small distance condition (Tukey-corrected p for all conditions: *p* ≤ 0.005).

## 4 Discussion

We examined the arithmetic relatedness and distance effects in multiplication fact retrieval in implicit and explicit mental arithmetic tasks. We aimed to clarify the functional significance of morphologically potentially similar ERP effects in both tasks. Uniquely, the same participants took part in both number-matching and arithmetic-verification tasks and the stimuli used in the two tasks were exactly the same.

### 4.1 The effect of task requirements on behavioral and ERP phenomena

Our first aim was to clarify whether functionally similar behavioral and ERP responses appear in both tasks. On the behavioral side, the number-matching task and the arithmetic-verification task showed clear differences. The number-matching task elicited the arithmetic relatedness effect for both reaction times and accuracy. In line with previous studies (Galfano et al., 2004, Rusconi et al., 2004), the participants were slower and less accurate in rejecting product probes than neutral probes. Unlike Galfano et al., 2003, Galfano et al., 2009, the arithmetic relatedness effect did not emerge in the comparison between multiples and neutral trials, although this may simply be due to the different criteria adopted for stimuli selection. On the contrary, the verification task showed only the distance effect, as participants were faster and more accurate in rejecting large distance than small distance probes, in line with Szűcs and Csépe (2005). The fact that the performance of the same group of participants resulted in different behavioral effects, may give a first hint about the different processes underlying arithmetic fact retrieval in the two tasks. As suggested by Galfano et al. (2003), in number-matching tasks, the implicit access to the arithmetic facts lexicon seems to rely on automatic activation that spreads from the operands to their products and, with less strength, to their multiples. Instead, when the arithmetic facts lexicon is accessed explicitly, retrieval is likely to be influenced by the distance of the numbers on a mental number line (Restle, 1970) and the representations of two close numbers overlap more which, in turn, makes discrimination more difficult.

On the electrophysiological side, although both tasks elicited similar components, ERPs in number matching and arithmetic verification showed different sensitivity to the different probe types, suggesting that the access to the arithmetic fact lexicon may be modulated by task requirements. The first aspect worth reporting is the fact that the effect of probe type was detected in different time windows. In the number-matching task, the effect of probe type was present only between 460 and 600 ms, whereas probe type yielded significant effects in three different time windows (260–312 ms, 379–452 ms and 600–752 ms) in the arithmetic verification task. This dissociation seems to suggest that both tasks involve semantic processes (N400 effect), but only the verification task seems to involve mechanisms related to the detection of expectation violation (N2b effect). The P3b was detected in both tasks. Its latency is thought to be an index of classification speed, which is proportional to the time required to detect and evaluate a target stimulus (Polich, 2007) and in the present study has been found to result in patterns that differed according to the task. The arithmetic relatedness effect on the P3b latencies was absent in the number-matching task. One explanation may be that neither product nor multiple probes were treated like hits, in line with the requirements of the number matching task. A second possibility is that P3b latencies were influenced by stimulus probability. Although each probe type was displayed the same number of times, the neutral probes (small distance and large distance probes together) were twice more likely to be displayed compared to product or multiple probes alone. This may have influenced our data according to the findings that relative frequent events have been found to elicit significantly shorter latencies compared to rare stimuli, probably because frequent stimuli maintain a stronger representation in memory (Polich & Margala, 1997). On the contrary, the clear arithmetic relatedness effect elicited in the verification task resulted from product trials eliciting a shorter latency than neutral and multiple trials, thus suggesting that less effort was put into the evaluation of product probes compared to neutral and multiples. This may be due to fact that, in the verification task, product probes were actually considered hits. We discarded the possibility that the difference between product probes and multiple probes resulted from a difference in activation in the arithmetic facts lexicon. This was suggested by the fact that multiple also elicited longer latencies than neutral probes and we interpreted it more like a distance effect than an arithmetic relatedness, effect due to stimuli selection.

As regards the distance effect, it was only found in the matching task. In light of Szűcs and Csépe's (2005) findings, we expected it to be elicited in the verification task also, but this was not the case for our study. However, looking at the mean values of latencies (Table 2), we can observe that in both tasks latencies elicited by small distance probe type were longer than latencies elicited by large distance probe types. Hence, perhaps the effect size was too small to detect.

**Fig. 7:**
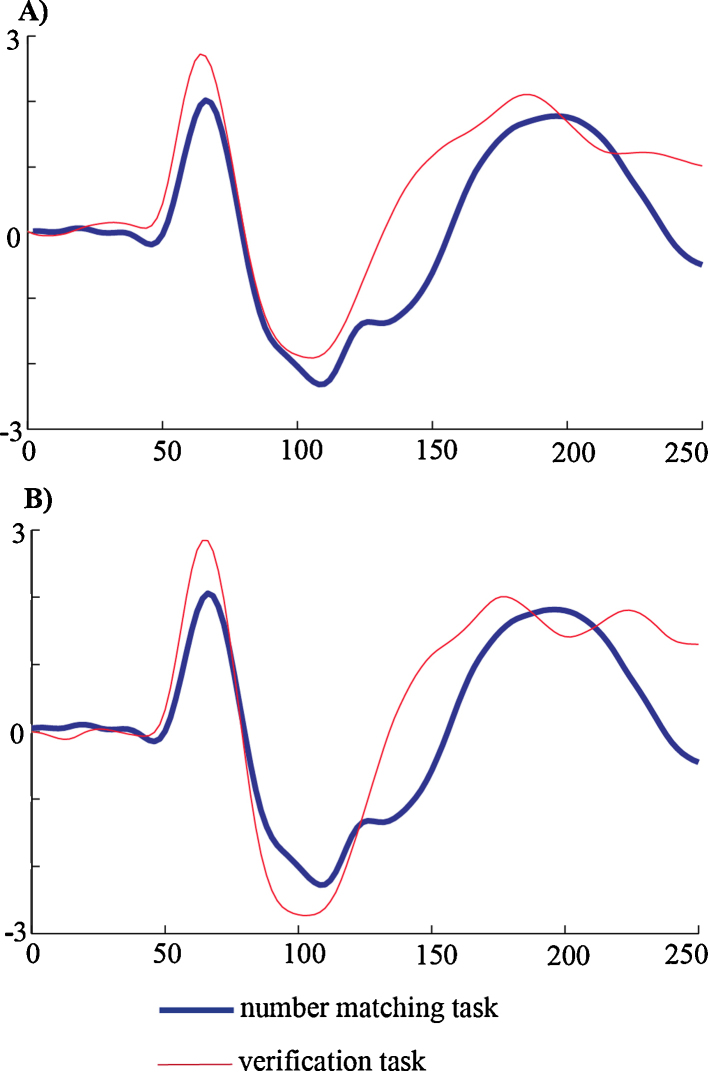
The P1 and N1 components elicited by the verification task and the matching task over left (A) and right (B) occipito-parietal sites. Note that positive voltage is upwards. X axis: time in milliseconds. Y axis: Voltage in μV.

Because we used a constant order in the task sequence (i.e., the number-matching task was always performed first), one may wonder whether the differences observed in the two tasks discussed above may simply reflect the effects of fatigue rather than differences in task demands. As argued in the methods section, we had to adopt this constraint in order to avoid transfer effects which might have prompted participants to voluntarily activate arithmetic knowledge when performing the number-matching task. In order to directly rule out the alternative account related to fatigue, we looked at early visual components of ERPs at occipito-parietal sites for the two tasks (see Fig. 7). Based on the notion that arousal and fatigue are inversely correlated (e.g., Dlugos et al., 2010), and on the fact that increased arousal results in a larger P1 at posterior sites (Vogel & Luck, 2000), we looked at P1 in the two tasks. Had fatigue played a crucial role in our data, we should have observed a smaller P1 for the arithmetic verification task, which was always performed second. Visual inspection clearly shows that the data, if any, suggest that P1 was actually more pronounced for the arithmetic-verification task than for the number-matching task. We also looked at difference in the N1 range, based on the observation that it has been reported that N1 amplitude seems to decrease as time on task and fatigue increases (Boksem, Meijman, & Lorist, 2005). Again, visual inspection suggests that no such effects were present in our data, as N1 amplitude was clearly larger for the arithmetic-verification task at least in right hemisphere sites. It is further to note that, if anything, the increased P1/N1 amplitudes in the verification task may point to increased attentional demands in this task, most probably because of its inherent properties. This observation is also incompatible with increased fatigue in the verification task. In conclusion, based on the analyses on early components, we can reasonably rule out the possibility that fatigue played any major role in our data and acted as a confound in our study. It is further to note that while the two tasks had dissimilar timings this is also unlikely to influence interpretation because absolute voltage levels were not compared across the tasks. Rather, we analyzed experimental effects relative to the task-specific baselines.

### 4.2 Arithmetic relatedness effect and distance effect

The second aim of the study was to investigate whether the arithmetic relatedness effect and the distance effect are sensitive to task requirements. As already mentioned above, behavioral data suggest that the two tasks may elicit the two effects differently. Implicit tasks seem to elicit mainly the arithmetic relatedness effect, while the verification task elicits a larger distance effect. However, ERP amplitudes showed that both effects are elicited by both tasks, but that their topography may vary according to task requirements. In the matching task, whereas the arithmetic relatedness effect appeared as a left frontal negativity and a right posterior positivity, the distance effect appeared as a right posterior negativity and left frontal positivity. Interestingly, the contrast between the product and the multiple conditions (Fig. 3) was similar to the product versus neutral probes contrast. According to Galfano et al. (2009), the activation of multiples is initially similar to the activation received by products, but it starts fading away at 350 ms after stimulus onset. Thus, our data suggest that the activation has already decayed to the level of activation of neutral probes by 460–600 ms.

The arithmetic relatedness N400 effect has usually been localized over central electrodes (e.g., Niedeggen & Rösler, 1999). We speculate that frontal effects here may be related to the fact that left frontal brain areas may be involved in semantic processes, in particular they are thought to mediate the selection of highly activated candidate representations (Lau, Phillips, & Poeppel, 2008). Moreover, the comparison between related and unrelated conditions showed stronger left posterior effects as well. This is in line with studies indicating that the multiplication lexicon would be localized in posterior areas of the left hemisphere (Cappelletti, Lee, Freeman, & Price, 2010). The observation that the distance effect was reflected in amplitude differences mainly over right posterior electrodes has also been reported in former studies and it has been attributed to automatic activation of number magnitude (Dehaene, 1996, Pinel et al., 2001). Thus, we suggest that implicit tasks mediate the access to representations of numerical magnitude (Cohen et al., 2000, Dehaene et al., 1996) along with semantic access to the memory lexicon. Our results are partially in contrast with those reported by Galfano et al. (2004) who found a right-lateralized arithmetic relatedness effect. This pattern is inconsistent with the hypothesis that the multiplication facts lexicon would mainly be localized in the left hemisphere. One possibility is that the lateralization of the arithmetic relatedness effect reported by Galfano et al. (2004) may be related to the fact that their crucial stimuli often showed a regular bias in magnitude, a feature that seems primarily processed in the right hemisphere (e.g., Dehaene, 1996). This aspect of stimuli selection was more controlled for in the present study.

The distance effect and the arithmetic relatedness effect were reflected in modulations detected over different areas in the arithmetic verification task. The arithmetic relatedness effect appeared mainly over right frontal areas in the 260–312 ms time window (N2b effect), it shifted over bilateral central areas between 379 and 452 ms (early N400 effect) and bilateral central-posterior areas between 600 and 752 ms (late N400 effect). The N2b is considered to be a correlate of covert orienting of attention, attentional capture and identification of stimuli. It appears in comparison tasks which require a decision on either physical or semantic characteristics of stimuli (Czigler and Csibra, 1992, Näätänen and Picton, 1986, Wijers et al., 1989). Indeed, it is a general correlate of detecting mismatch between the representations of task-relevant features. Given that the verification task employed in the present study is similar to the one implemented by Szűcs and Csépe (2005), it may be that our N2b is equivalent to the N3 component investigated in their study and that such components are related to the detection of expectation violation. Also, the latency of the N2b has been found to reflect the distance effect in verification tasks (Szűcs & Csépe, 2005). In the present study, small distance probes elicited longer latencies than large distance probes. According to Szűcs and Csépe (2005) this could reflect the distance-differential speed of discrimination of correct vs. incorrect activation patterns on the mental number line. It is evident that the N400 effect was detected over different electrodes compared to the matching task. One explanation could be that in the arithmetic-verification task, attention plays a greater role and that this may affect the N400 effect topography (for full review, see Kutas & Federmeier, 2011). Szűcs and Csépe (2005) demonstrated that attentional mechanisms influence the posterior topography of the N400 effect. In their study, the stimulus probability was manipulated and probes belonged to one of three conditions: the proportion of incorrect probes could be 20%, 50% or 80%. Results showed that an occipital shift of the distribution of the N400 effect could be seen in the 20% and 80% conditions relative to the 50% condition. Since an increased negativity of the amplitude of the occipital N2 around 200–300 ms has been described as increased discrimination load in visual search and in semantic content analysis (Eimer, 1996), they concluded that the posterior localization of the N400 effect could be explained by the influence of attention-related posterior ERPs. Hence, we hypothesized that in the present study the different topography in the two tasks may be due to the different role that attentional mechanisms play in the matching and the verification tasks.

Indeed it is reasonable to posit that data in the number-matching task were not influenced by strategic factors, while performance in the arithmetic-verification task was likely to have been influenced by goal-directed mechanisms.

It is noteworthy that in the arithmetic verification task the variation of the amplitude reflecting the distance effect was found in left-parietal parts of the scalp. Neuropsychological and neuroimaging evidence showed the ERP correlate of the distance effect mainly over right areas (e.g., Dehaene, 1996), which may be dedicated to the mental manipulation of numerical quantities. In contrast, left posterior areas are associated to the verbal code according the triple-code model (Dehaene & Cohen, 1996). In such a verbal code, numbers are represented as a parsed sequence of words. This representation is the primary code for accessing a rote verbal memory of arithmetic facts (e.g., “three times five, fifteen”). Operands of the problem (3 × 5) are transcoded into a verbal representation (“three times five”) which is then used to trigger completion of this word sequence using rote verbal memory (“three times five, fifteen”) (Dehaene & Cohen, 1997). The sensibility of left posterior areas to the distance effect may be explained by incorrect results experienced through life and especially during school. Indeed, incorrect answers close to the correct result are more likely to be experienced than incorrect answers far from the correct result, hence resulting in a stronger representation in verbal memory. The failure of previous studies to obtain similar results may be due to the fact that they did not assess the distance effect through the multiplication verification task, which is known to have a strong connection to verbal memory. If this finding is confirmed by future research, we would have provided evidence that the verification task, at least with multiplication, is not only influenced by attentional-driven mechanisms but also by rote verbal memory.

### 4.3 The impact of implicit and explicit access to numerical knowledge on the N2b and the N400

The third aim of the present study was to clarify whether the N2b and the N400 are elicited irrespective of whether arithmetic and numerical knowledge are accessed explicitly and are critical for the task at hand. The N400 effect was elicited and influenced by probe type in both tasks. This suggests that semantic processes are involved either when arithmetic facts are accessed explicitly or implicitly. Although the N2b was elicited in both tasks, it was sensitive to probe type only in the verification task. The amplitude of the N2b is thought to be a correlate of detection of expectation violation and it is elicited by task relevant features (Folstein & Van Petten, 2008). In arithmetic-verification tasks, participants are explicitly asked to perform arithmetic, therefore they have to mentally compute the operation. Then, when it comes to compare the computed result to the one presented on the screen, probes other than products represent a mismatching stimulus. On the contrary, in the number-matching task participants are not explicitly required to perform arithmetic. In this case, the participants do not compute any operation and all non-matching probes are likely to be considered non-target stimuli, irrespective of their arithmetic relation to the cue or the distance from the product. In other words, the lack of a modulation as a function of probe type in the number-matching task may simply reflect the fact that the expectation violation was detected similarly irrespective of the specific experimental condition. In sum, the present data are consistent with the view that the N2b effect seems to be more related to goal-directed rather than to automatic facts retrieval.

## 5 Conclusion

We compared access to the arithmetic lexicon using both a task where arithmetic was task-irrelevant and a task where arithmetic was task-relevant. Although the literature identified the same ERP components in both tasks, our results suggest that different processes underlie the access to the arithmetic facts lexicon. When arithmetic facts are accessed explicitly, the detection of violation of the expectation is activated along with semantic processes, while during implicit access only semantic processes seem to play a key role. Secondly, we provide evidence that the arithmetic relatedness effect and the distance effect are sensitive to task requirements. Finally, we clarified that the N2b and the N400 are also modulated by task requirements. While the N400 is sensitive to different experimental conditions in both tasks, the N2b seems to be linked to goal-directed mechanisms involved in explicit tasks.

## Figures and Tables

**Table A.1:** Stimuli in the non-matching and matching probe types used in the number-matching task.

Product	Multiple	±2 Small distance	±4 Large distance	Fillers
3·5–15	3·5–10	3·5–17	3·5–19	67·8–42
6·3–18	6·3–57	6·3–20	6·3–14	3·72–56
3·9–27	3·9–63	3·9–29	3·9–23	26·7–45
6·9–54	6·9–24	6·9–52	6·9–50	4·53–37
9·8–72	9·8–16	9·8–74	9·8–76	53·9–12

Product probe balancing	Multiple probe balancing	±2 SD probe balancing	± 4 LD probe balancing	Cue balancing
4·15–15	10·4–10	17·5–17	19·5–19	3·5–5
18·7–18	6·57–57	3·20–20	3·14–14	6·3–3
8·27–27	63·7–63	29·6–29	23·5–23	3·9–9
54·5–54	9·24–24	8·52–52	9·50–50	6·9–6
5·72–72	16·3–16	74·9–74	76·8–76	9·8–8

**Table A.2:** Stimuli used in the arithmetic-verification task.

Product	Multiple	±2 Small distance	±4 Large distance
3 × 5 = 15	3 × 5 = 10	3 × 5 = 17	3 × 5 = 19
6 × 3 = 18	6 × 3 = 57	6 × 3 = 20	6 × 3 = 14
3 × 9 = 27	3 × 9 = 63	3 × 9 = 29	3 × 9 = 23
6 × 9 = 54	6 × 9 = 24	6 × 9 = 52	6 × 9 = 50
9 × 8 = 72	9 × 8 = 16	9 × 8 = 74	9 × 8 = 76
